# Alzheimer's disease patients have smaller venous drainage system compared to cognitively healthy controls

**DOI:** 10.1002/alz.14551

**Published:** 2025-02-12

**Authors:** Keshet Pardo, Vadim Khasminsky, Ophir Keret, Felix Benninger, Ilan Goldberg, Ilan Shelef, Eitan Auriel, Amir Glik

**Affiliations:** ^1^ Department of Neurology Rabin Medical Center – Beilinson Hospital Petach Tikva Israel; ^2^ School of Medicine Tel‐Aviv University Tel‐Aviv Israel; ^3^ Department of Radiology Rabin Medical Center – Beilinson Hospital Petach Tikva Israel; ^4^ Cognitive Neurology Service Rabin Medical Center – Beilinson Hospital Petach Tikva Israel; ^5^ Department of Neurology Icahn School of Medicine at Mount Sinai New York New York USA; ^6^ Department of Radiology Soroka Medical Center Beer Sheva Beersheba Israel

**Keywords:** Alzheimer's disease, cognitive impairment, veins, venous drainage

## Abstract

**INTRODUCTION:**

One of the pathological hallmarks of Alzheimer's disease (AD) is the accumulation of amyloid beta 42 (Aβ42). Decreased venous drainage may enhance Aβ42 accumulation. We aimed to compare venous cross‐sectional area (CSA) of AD patients to cognitively healthy controls.

**METHODS:**

All patients underwent neurocognitive evaluation and brain magnetic resonance imaging, including time‐of‐flight sequence. Venous CSA was measured at the jugular foramen level.

**RESULTS:**

Thirty‐nine AD/mild cognitive impairment patients and 20 cognitively healthy controls were included. Total venous CSA was smaller in the cognitively impaired group (mean CSA 139.77 mm^2^ [SD: 32.22] vs 166.55 mm^2^ [SD: 33.1], *p* = 0.004]. When divided, both internal jugular and non‐jugular systems were smaller within cognitively impaired patients; statistical significance was achieved only for the non‐jugular system (mean CSA 41.21 mm^2^ [SD: 21.52] vs 54.5 mm^2^ [SD: 27.31], *p* = 0.045).

**DISCUSSION:**

There is an association between smaller venous systems and cognitive impairment, most prominently in the non‐jugular system. Venous narrowing may cause impaired venous drainage, leading to an accumulation of Aβ42.

**Highlights:**

- The non‐jugular venous system, including the vertebral plexus and pterygopalatine plexus, plays an important role in cerebral drainage.
- The total venous CSA is significantly smaller in cognitively impaired patients compared to healthy controls.
- Reduced venous drainage may contribute to the accumulation of Aβ and other waste products and potentially plays a role in AD pathology.

## 1 BACKGROUND

Alzheimer's disease (AD) is the world's leading cause of dementia and the fifth leading cause of death in the US.^1^ As the average life expectancy increases, the prevalence of dementia also increases.^2^ The pathologic changes in AD include plaques composed of amyloid beta 42 (Aβ42), tangles composed of hyperphosphorylated tau, and inflammatory responses and brain atrophy.^3^ Additionally, vascular factors, including vascular risk factors,^4^ are associated with cognitive decline in AD patients.^5^, ^6^ The clinical presentation of AD includes various cognitive impairments and is preceded by a prolonged asymptomatic period during which the pathological changes are already present.^7^, ^8^ One of the pathological processes active during the first stages of the disease is the accumulation of Aβ42 in the central nervous system (CNS),^9^ and it has been shown that an imbalance between the formation and clearance rates leads to the accumulation of AΒ42, which causes neuronal damage.^10^ The rate of Aβ42 clearance in AD patients is decreased by about 30% compared to the normal population, as demonstrated in previous work.^11^

**FIGURE 1 alz14551-fig-0001:**
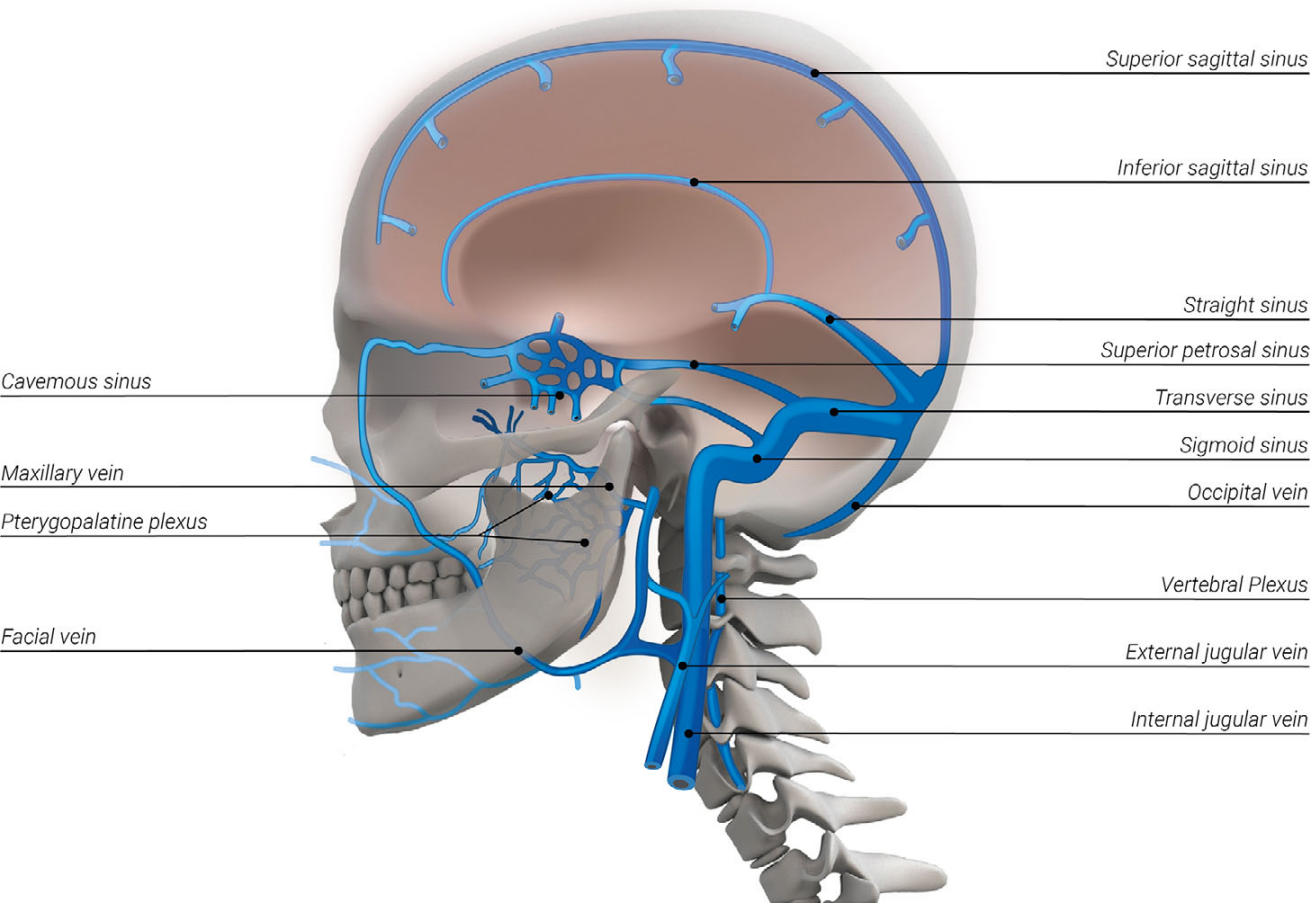
Cerebral venous system.

The current vein‐related mechanism of CNS waste product removal relies on proteins being transferred to the cerebrospinal fluid (CSF) and through the arachnoid granulations to the cerebral venous sinuses.^12^, ^13^ The cerebral venous sinuses are then drained via two main systems: the internal jugular vein (IJV) system and the non‐jugular vein (NJV) system, which is composed of the vertebral plexus and pterygopalatine plexus^14^ (Figure 1). While the IJV is usually the more prominent of the two, it is reported that for 6% of the population, the NJV is the more prominent cerebral drainage system.^15^ Both the IJV and NJV undergo postural changes and reduced drainage in the standing position compared to the supine position.^16^ However, NJV drainage reduction is less prominent in the standing position compared to the IJV; as such, the NJV might play a more significant role while standing.^15^

Prior work identified a relationship between venous drainage abnormalities and several neurologic disorders, as well as advanced age.^17^ The low clearance rate of CNS waste products due to decreased venous drainage has been suggested as a contributing factor to the pathology of neurodegenerative diseases,^18^ a hypothesis that is also supported by an animal AD model with a venous defect.^19^ Insufficiency of the jugular veins was linked to periventricular white matter changes in AD patients, further supporting venous outflow impairment as one of the factors in the disorder.^20^

A previous study from our group successfully quantified the cross‐sectional area (CSA) of the IJV and NJV systems in a healthy, age‐variable population, relying on a time‐of‐flight (TOF) MRI sequence, demonstrating a smaller NJV system in the older population.^21^ In comparison, the CSA of the IJV was reported to be larger in the older population^22^ and found to be enlarged in association with decreased brain volume in an otherwise healthy population.^23^

**RESEARCH‐IN‐CONTEXT**

1. **Systematic review**: The venous system has recently attracted increased attention for its role in cerebral blood flow drainage and its impact on neurodegenerative diseases, including AD. It is suggested that impaired venous drainage may play a crucial role in the pathology of AD, possibly through the accumulation of substances such as Aβ42.
2. **Interpretation**: Our study found that both the internal jugular veins and non‐jugular veins were significantly narrower in patients with MCI and AD compared to healthy controls. A reduced venous CSA could indicate impaired drainage and a lower clearance rate of Aβ42 and other neurotoxic substances. Given the close interplay between the glymphatic and venous systems – where CSF waste is ultimately drained into the veins – dysfunction in either system can exacerbate the accumulation of Aβ42. This suggests that impaired venous drainage may contribute to glymphatic inefficiency, creating a vicious cycle that accelerates neurodegeneration.
3. **Future directions**: Further research is needed to explore this vascular aspect of neurodegenerative disease and its potential for developing new treatment strategies.

In this study, we aimed to measure the CSA of the jugular and non‐jugular venous drainage systems in AD demented and mild cognitive impairment (MCI) patients (cognitively impaired group) and compare it to a cognitively healthy (CH) cohort in the same age group. Our hypothesis was that the cognitively impaired group would have a smaller drainage system compared to the controls.

## 2 METHODS

### 2.1 Patient population

The AD demented and MCI patients were ascertained from the HORIZON 2020 Mes‐Cobard cohort, an ongoing deep phenotyping study intended to better describe neurodegenerative disease characteristics in the cognitive neurology clinic at Rabin Medical Center. Inclusion criteria were age 60 and above, a Montreal Cognitive Assessment (MoCA) score of up to 24,^24^ and a diagnosis of MCI or AD dementia according to the National Institute on Aging (NIA) 2011 guidelines.^25^ We excluded patients with dementia of other origins and patients with structural brain abnormalities. Cognitive assessments were made by neuropsychologists, and the diagnosis was determined by the consensus of two neurocognitive experts (A.G. and O.K.). The control group was composed of healthy cognitive subjects with no history of neurologic disease, as described in a previous study by our group.^21^, [Fig alz14551-fig-0001]

### 2.2 Magnetic resonance image acquisition

MRI was performed with a 3T scanner (Siemens Magnetom Prisma). Venous system demonstration was obtained by a 2D TOF, multisection sequence. The sections were acquired in a sequential single‐section mode, using a spoiled fast‐field echo with an inferiorly placed presaturation slab to eliminate the signal from arterial blood. The scanning plane was set parallel to the hard palate and localized on the midsagittal survey. Scan parameters were as follows: repetition time: 21.0 ms; time to echo: 3.0 ms; and section width, 0.7 mm with a 20% slice overlap. Voxel size was 0.3 × 0.3 × 0.7 mm, signal to noise ratio: 1.00. Volume reconstructions were performed using maximal intensity projections on the Extended Workspace workstation. Control group MRI scan acquisition was essentially similar and is described in a previous study by our group.^21^

### 2.3 Venous CSA measurements

**FIGURE 2:**
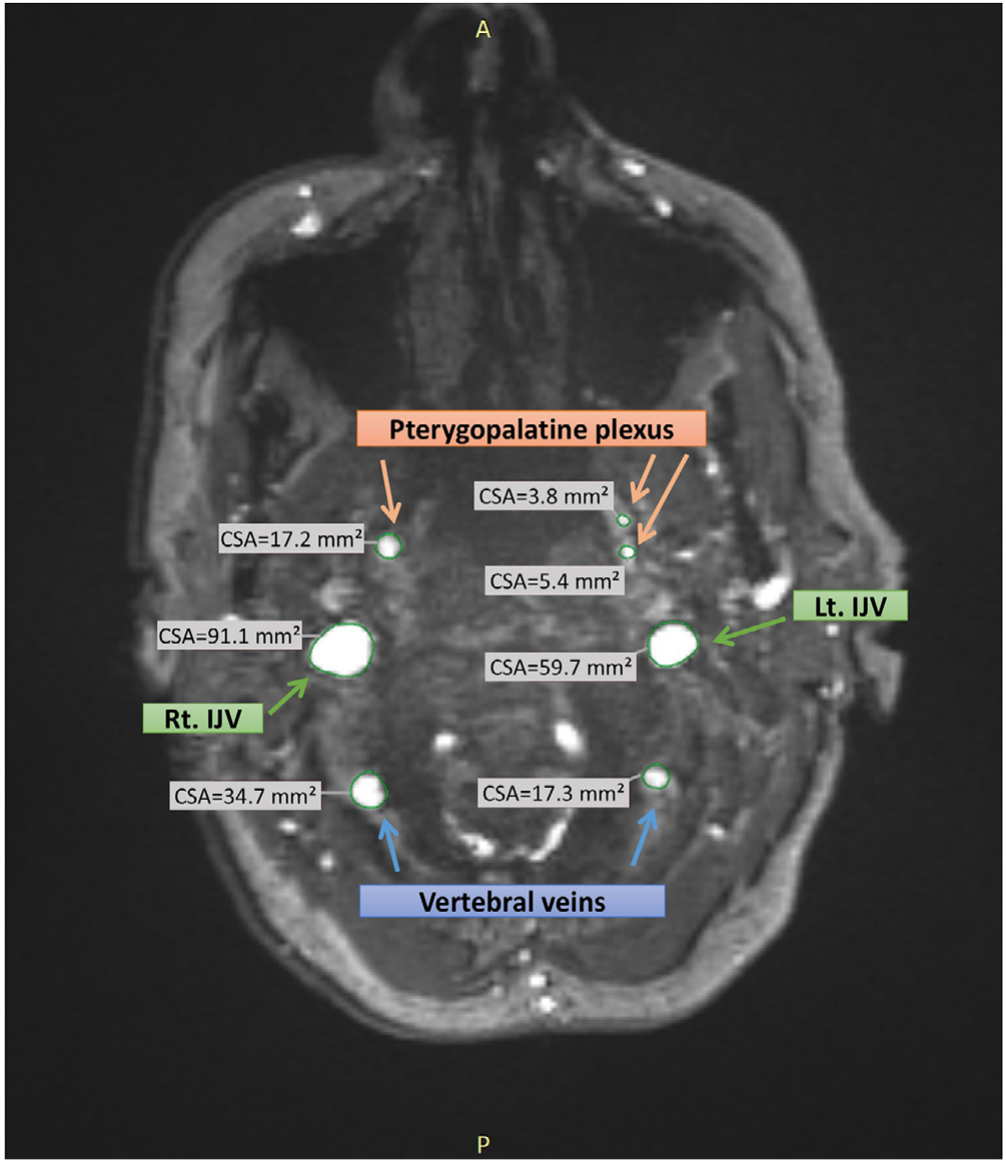
Measurement of cross‐sectional area using MRI time‐of‐flight sequence at level of jugular foramen. IJV, internal jugular vein; CSA, cross‐sectional area.

Venous measurements were made as described previously by our group.^21^ Venous CSA was measured at the level of the jugular foramen due to the vertical position of the veins at this level, enabling a precise and reliable CSA measurement. For each patient, we measured the CSA of the IJVs and NJVs, including the (1) pterygopalatine plexus, defined as all the veins draining the intracranial compartment, anterior to the IJV, and (2) vertebral veins of the vertebral plexus, defined as all the veins draining the intracranial compartment, posterior to the IJV. All measurements were bilateral. Superficial veins that are not part of the parenchyma drainage system were excluded. All the measurements were done by an experienced neuroradiologist (V.K.) who was blinded to the subjects diagnosis. Figure 2 presents a CSA measurement example.

### 2.4 Statistical analysis

Statistical analyses were performed using IBM SPSS Statistics for Windows, version 25.0 (IBM Corp., Armonk, NY, USA). Qualitative data were presented as frequencies and percentages, while quantitative data were presented as median and interquartile range (IQR) or mean and standard deviation, depending on the normality of the parameters. The Pearson chi‐squared test was used for the comparison of qualitative baseline characteristics, and the median test was used for non‐normally distributed data, including cognitive test scores. The *t*‐test was used for comparing venous CSA measurements, and one‐way ANOVA was used for comparing intergroup differences. The normality distribution of venous CSA measurements was assessed using the Kolmogorov–Smirnov test. Results were considered significant at a level of *p* < 0.05.

### 2.5 Statement of ethics

The study was approved by the local ethics committee (RMC‐0345‐21). Due to the retrospective non‐interventional design of the study, informed consent was not required.

The study followed observational cohort guidelines, according to Strengthening the Reporting of Observational Studies in Epidemiology (STROBE).

## 3 RESULTS

Sixty‐five patients were reviewed for the study: 20 cognitively healthy and 45 cognitively impaired patients. Six of the cognitively impaired patients were excluded due to poor MRI quality. A total of 39 cognitively impaired patients were included in the analysis, consisting of 20 patients with an AD dementia diagnosis and 19 patients with an MCI diagnosis. The median age was 68.3 years (IQR: 65.6 to 72.2), and 51.6% were female. The median MoCA score was 21 (IQR: 15 to 23), and the median Mini‐Mental State Examination score was 25 (IQR: 21 to 28). Table 1 presents baseline characteristics.

**TABLE 1:** Patient baseline characteristics (*n* = 39).

Age, median (IQR)	63.8 (65.6 to 72.2)
Female, *N* (%)	20 (51.6)
MoCA, median (IQR)	21 (15 to 23)
MMSE, median (IQR)	25 (21 to 28)
MCI, *N* (%)	19 (48.7)
Dementia, *N* (%)	20 (51.3)
Hypertension, *N* (%)	12 (30.8)
Dyslipidemia, *N* (%)	17 (43.6)
Diabetes, *N* (%)	10 (25.6)
Depression, *N* (%)	5 (13.2)

*Note*: Baseline characteristics of cognitively impaired group.

Abbreviations: MMSE, Mini‐Mental State Examination; MoCA, Montreal Cognitive Assessment.

The cognitively healthy group included 20 subjects, with a median age of 64 years (IQR: 62 to 69.5), which was not statistically different from the age of the cognitively impaired group; 60% were female.

### 3.1 Venous CSA measurements

The CSA of the venous drainage system (IJV and NJV together) was smaller in the cognitively impaired group compared to the cognitively healthy group (mean CSA 139.77 mm^2^ [SD: 32.22] vs 166.55 mm^2^ [SD: 33.1], respectively; *p* = 0.004). When dividing the venous drainage system into components, the mean IJV CSA was 98 mm^2^ [SD: 30.2] vs 112 mm^2^ [SD: 32.2] in the cognitively impaired and cognitively healthy groups, respectively, without achieving statistical significance. The mean NJV CSA was 41.21 mm^2^ [SD: 21.52] vs 54.5 mm^2^ [SD: 27.31] for the cognitively impaired and cognitively healthy groups, respectively; *p* = 0.045. Table 2.

**TABLE 2:** Venous CSA measurements.

	Cognitively impaired *n* = 39	Cognitively healthy *n* = 20	*p*
Total venous CSA (mm^2^), mean (SD)	139.77 (32.2)	166.55 (33.1)	0.004
IJV CSA (mm^2^), mean (SD)	98.56 (31.1)	112.05 (32.3)	0.118
NJV CSA (mm^2^), mean (SD)	41.21 (21.5)	54.5 (27.3)	0.045

Abbreviations: CSA, cross‐sectional area; IJV, internal jugular vein; NJV, non‐jugular vein; SD, standard deviation.

**FIGURE 3:**
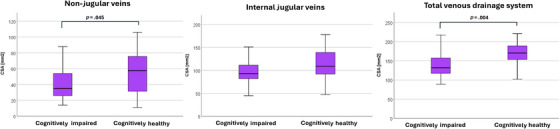
Cross‐sectional area measurements of cerebral veins (mm^2^). CSA, cross‐sectional area.

**FIGURE 4:**
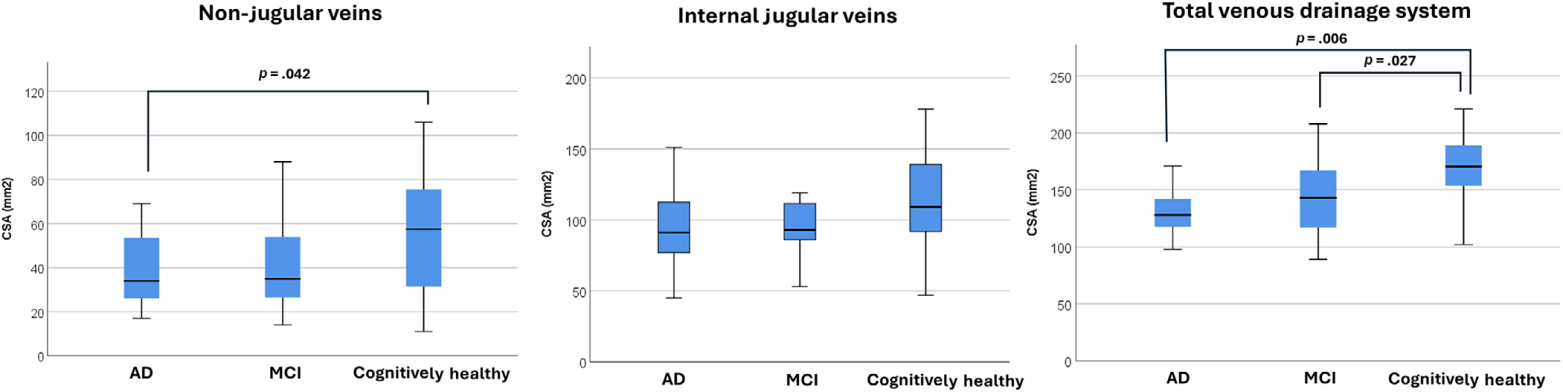
Cross‐sectional area measurements of cerebral veins (mm^2^). CSA, cross‐sectional area.

We also performed a subanalysis, comparing the measurements between the AD, MCI, and cognitively healthy groups. The mean total CSA was smaller in the AD group (136.9 mm^2^ [SD: 32.83]) compared to the MCI group (142.79 mm^2^ [SD: 32.18]) and the cognitively healthy group (166.55 mm^2^ [SD: 33.1]; *p* = 0.014); Table 3, Figures 3 and 4).

**TABLE 3:** Venous CSA measurements, subdivision of cognitively impaired group.

	Dementia, *n* = 20	MCI, *n* = 19	Cognitively healthy, *n* = 20	*p*
Total venous CSA (mm^2^), mean (SD)	136.9 (32.8)	142.79 (32.2)	166.55 (33.1)	0.014
IJV CSA (mm^2^), mean (SD)	98 (32.7)	99.16 (28.2)	112.05 (32.3)	0.297
NJV CSA (mm^2^), mean (SD)	38.9 (17.5)	43.63 (25.4)	54.5 (27.3)	0.113

Abbreviations: CSA, cross‐sectional area; IJV, internal jugular vein; MCI, mild cognitive impairment; NJV, non‐jugular vein; SD, standard deviation.

### 3.2 Sex difference in venous CSA measurements

Cognitively impaired females had smaller CSAs of both IJVs and NJVs compared to males (mean IJV CSA 99.06 mm^2^ [SD: 27.74] vs 102.27 mm^2^ [SD: 36.2], respectively; and mean NJV CSA 36.44 mm^2^ [SD: 17.84] vs 46.73 mm^2^ [SD: 24.15], respectively); however the difference was not statistically significant. Cognitively healthy females had a larger IVJ CSA compared to males (mean IJV CSA 123.08 mm^2^ [SD: 26.56] vs 95.50 mm^2^ [SD: 34.8], respectively), while a smaller NJV CSA compared to males (mean NJV CSA 45.33 mm^2^ [SD: 21.03] vs 68.25 mm^2^ [SD: 31.13], respectively), without reaching statistical significance (supplemental material, Table S1). Comparing the cognitive healthy group to dementia and MCI, females with dementia had smaller mean IJV CSAs compared to males, and females with MCI had smaller NJV CSAs compared to males (supplemental material, Table S2).

### 3.3 Differences in venous CSA between right and left venous systems

We compared the measurements of the right and left venous CSAs. The comparison was done both for the entire cohort and after subdividing the cohort into dementia, MCI, and cognitively healthy groups. The CSA of the right jugular venous system was significantly larger than the left jugular venous system for the entire cohort (mean right IJV CSA of 63.1 mm^2^ [SD: 30.86] compared to left IJV CSA 40.03 [SD: 23.94], *p* < 0.001), and this difference remained consistently significant within the cognitive impaired groups as well but didn't reach statistical significance in the cognitively healthy group. There was no significant difference between the right and left NJV CSA in all groups (Table 4).

**TABLE 4:** Comparison between right and left venous drainage systems.

CSA (mm^2^), mean (SD)	Right	Left	*p* value^*^
**IJV**			
**Entire cohort**	63.1 (30.86)	40.03 (23.97)	<0.001
Dementia	60.85 (30.45)	37.15 (21.13)	0.018
MCI	66.26 (25.45)	32.89 (15.65)	<0.001
Cognitively healthy	62.35 (36.75)	49.7 (30.3)	0.53
**NJV**			
**Entire cohort**	22.78 (15.01)	22.68 (14.55)	0.964
Dementia	19.55 (9.95)	19.35 (9.14)	0.909
MCI	21.42 (13.5)	22.21 (14.17)	0.76
Cognitively healthy	27.3 (19.54)	26.68 (14.55)	0.89

Abbreviations: CSA, cross‐sectional area; IJV, internal jugular vein; MCI, mild cognitive impairment; NJV, non‐jugular vein; SD, standard deviation.

* Paired *t*‐test.

### 3.4 Cardiovascular risk factors and periventricular white matter disease

We found no significant difference in venous CSA measurements between cognitively impaired patients with and without cardiovascular risk factors (supplemental material, Tables S3 and S4).

Additionally, we found no significant differences in venous CSA measurements between cognitively impaired patients with and without periventricular white matter disease, as measured by white matter hyperintensity (WMH) (supplemental material, Table S5).

## 4 DISCUSSION

Our study demonstrates an association between smaller venous CSA and cognitive impairment due to AD and MCI, with the most pronounced association observed in the NJV system. The association between impaired venous drainage and AD has become clearer recently; however, only a few studies have demonstrated it directly. Cerebral periventricular WMHs among AD patients are associated with venous insufficiency, expressed as jugular venous reflux. This finding suggests that the venous system is involved in AD pathology.^20^ Similar venous changes have been observed in aging and linked to cerebral hypoperfusion, drainage disruption, and vasogenic edema^26^ in both AD and elderly subjects.^27^

AD patients have a reduced Aβ42 clearance rate without a change in the production rate, as shown by metabolic labeling,^11^ which leads to aggregation linked with neuronal damage.^17^ The association between venous insufficiency and decreased clearance of waste products, such as Aβ42, can be explained by several mechanisms. One possible explanation is the relationship between venous outflow and CSF dynamics. This theory is supported by studies showing changes in CSF dynamics due to venous drainage insufficiency,^28^ including a correlation between extracranial venous CSA and CSF pulsatility in both healthy controls and multiple sclerosis patients.^29^

Another possible link between venous insufficiency and decreased clearance of CNS waste products is through the glymphatic system. The glymphatic system facilitates waste removal within the CNS via perivascular spaces.^30^ Its efficiency is diminished in cases of venous insufficiency.^17^, ^31^ Impairment of the glymphatic system leads to decreased Aβ42 clearance and its subsequent accumulation in AD patients, resulting in cognitive decline.^32^, ^33^, ^34^ Furthermore, animal models have demonstrated reversible impairments in both brain perfusion and cognitive function due to changes in the glymphatic system.^35^ Glymphatic vessels are difficult to visualize using standard tools; however, they are known to surround cerebral veins.^34^ It is possible that smaller venous CSA serves as a marker for dysfunction in both the venous and glymphatic systems.

Finally, an additional mechanism explored in relation to AD and venous dysfunction is the deposition of amyloid within veins. Such deposits may lead to venous collagenase activity, impair cerebrovascular pulsations, and enlarge perivascular spaces, all of which contribute to waste clearance dysfunction.^36^ Venous collagenosis as a cause of venous insufficiency and stenosis has also been reported in the deep medullary venous system, manifested by periventricular WMH.^37^, ^38^, ^39^ This slow, progressive process leads to leakage around the venules and vasogenic edema^40^ and is exacerbated with age and cardiovascular risk factors.^41^ WMHs, as mentioned earlier, are linked to cognitive impairment in AD patients, and an association between WMH disease and impaired amyloid clearance in AD patients has also been described.^42^ Our study did not establish an association between smaller CSA of the IJVs or NJVs and periventricular WMHs. Perhaps the pathological process in the veins measured in our cohort is somewhat different, or a larger cohort is needed.

Inter‐hemispheric differences in CSA of the jugular veins are known, with larger CSA of the right‐sided IJVs described in previous studies^43^ and believed to be associated with larger dural venous sinuses in the right hemisphere,^44^ as well as with the relatively straight drainage path of the right IJV into the superior vena cava, thereby reducing blood flow resistance. This has also been referenced in studies concerning cannulation of the IJVs.^45^ Our study was in line with previous reports demonstrating larger right IJV CSA; however, the difference was mostly pronounced within the cognitively impaired group. This could be explained by the left IJV being more susceptible to injury, thereby causing a more pronounced difference within cognitively impaired patients, but further research should be done to determine the cause.

Unlike most studies that primarily describe changes in IJVs, our study quantified both the jugular and non‐jugular systems, providing a comprehensive view of venous cerebral drainage. We found that the differences in CSA size between the cognitively impaired and healthy populations were more pronounced in the NJV system. Specific changes in the non‐jugular system have been described sporadically in AD^46^ and aging^21^; our findings support the notion that the NJV system plays an important role in waste clearance and possibly in AD pathophysiology.

Our study also demonstrated differences in the venous CSA of females compared to males, with smaller venous CSAs in cognitively impaired females compared to males, which was not the case in the cognitively healthy group. Few studies have been conducted on this topic, but we believe it might coincide with the higher percentage of females diagnosed with AD.

The limitations of our study include its retrospective nature and the use of a historical cohort as the control group, which may introduce measurement bias due to differences in MRI machines. However, since the sequence used and the magnetic field strength were the same, we anticipate that any differences would be minor, if present at all. An additional limitation of our study is the lack of use of proteomics to assess the glymphatic system or Aβ42 clearance.

In conclusion, our study demonstrates an association between a smaller CSA of the venous drainage system and both AD dementia and MCI, likely due to a reduced clearance rate of Aβ42 and other inflammatory or waste products. Aβ has recently become a target for treating and delaying further cognitive decline in AD patients.^47^ Further benefits could be achieved by improving our understanding of the mechanisms behind amyloid production–clearance mismatch^48^ while exploring ways to prevent decreased clearance rates due to venous insufficiency.

## CONFLICT OF INTEREST STATEMENT

The authors report no competing interests. Author disclosures are available in the supporting information.

## CONSENT STATEMENT

Due to the retrospective non‐interventional design of the study, informed consent was not required.

## Supporting information



Supporting Information

Supporting Information

Supporting Information

## Data Availability

The data that support the findings of this study are available from the corresponding author, upon reasonable request.
