# Supplementary material for: Alzheimer's disease patients have smaller venous drainage system compared to cognitively healthy controls
Source: Alzheimers Dement. 2025 Feb 12;21(2):e14551. doi: 10.1002/alz.14551 (PMC11851167; doi:10.1002/alz.14551)
Supplement: Supplementary file 1 — Supporting Information [file ALZ-21-e14551-s003.pdf]

Supplemental materials:

Sex differences:

Table – S1

Venous CSA measurements, comparison by sex

	**Cognitive impaired**			**Cognitive Healthy**		
	Male	Female	P value	Male	Female	P value
Total venous CSA [mm^2], mean (SD)	149 (36.18)	135.5 (30.68)	0.271	163.75 (47.29)	168.42 (21.41)	0.766
IJV CSA [mm^2], mean (SD)	102.27 (36.2)	99.06 (27.74)	0.783	95.50 (34.8)	123.08 (26.56)	0.059
NJV CSA [mm^2], mean (SD)	46.73 (24.15)	36.44 (17.84)	0.186	68.25 (31.13)	45.33 (21.03)	0.064

MCI, Mild cognitive impairment; CSA, Cross sectional area; IJV, Internal Jugular veins; NJV, Non-jugular veins

Table – S2

Venous CSA measurements, subdivision of cognitive impaired group and sex

	**Dementia**			**MCI**			**Cognitive Healthy**		
	Male	Female	P value	Male	Female	P value	Male	Female	P value
Total venous CSA [mm^2], mean (SD)	143.63 (35.28)	133.38 (37.8)	0.580	155.14 (40)	137.63 (24.07)	0.307	163.75 (47.29)	168.42 (21.41)	0.766
IJV CSA [mm^2], mean (SD)	105.5 (36.55)	92.63 (32.42)	0.470	98.57 (38.33)	105.5 (22.44)	0.671	95.5 (34.8)	123.08 (26.56)	0.059
NJV CSA [mm^2], mean (SD)	38.13 (13)	40.75 (21.55)	0.770	56.57 (30.85)	32.13 (13.24)	0.062	68.25 (31.13)	45.33 (21.03)	0.064

MCI, Mild cognitive impairment; CSA, Cross sectional area; IJV, Internal Jugular veins; NJV, Non-jugular veins

Cardiovascular risk factors:

Table – S3

Cardiovascular risk factors summary within Cognitive impaired group

Num of risk factors	n (%)
None	15 (38.5)
1	13 (33.3)
2	7 (17.9)
3	4 (10.3)

Risk factors: Diabetes, hypertension and hyperlipidemia

Table – S4

Cardiovascular risk factors and venous CSA measurements within the cognitive impaired group

	No cardiovascular risk factors	At least one cardiovascular risk factor	P value
	n=15	n=24	
Total venous CSA [mm^2], mean (SD)	137.27 (38.27)	141.33 (28.58)	0.707
IJV CSA [mm^2], mean (SD)	97.93 (34.68)	98.96 (27.82)	0.919
NJV CSA [mm^2], mean (SD)	39.33 (20.25)	42.38 (22.63)	0.673

Risk factors: Diabetes, hypertension and hyperlipidemia
CSA, Cross sectional area; IJV, Internal Jugular veins; NJV, Non-jugular veins

Table – S5

**Periventricular white matter and venous CSA measurements within cognitive impaired group**			
	No WMH	WMH	P value
	n = 13	n = 26	
Total venous CSA [mm^2], mean (SD)	135.15 (25.33)	142.08 (35.4)	0.713
IJV CSA [mm^2], mean (SD)	96 (22.39)	99.85 (33.76)	0.680
NJV CSA [mm^2], mean (SD)	39.15 (24.28)	42.23 (20.44)	0.534

WMH, white matter hyperintensity; CSA, Cross sectional area; IJV, Internal Jugular veins; NJV, Non-jugular veins
